# Central venous catheter–related right atrial thrombus in oncology patients: a case series of cardiovascular magnetic resonance studies

**DOI:** 10.1093/ehjcr/ytae296

**Published:** 2024-06-28

**Authors:** Isabel Cardoso, José de Almeida, Zoi Tsoumani, Francisco Alpendurada, Raad H Mohiaddin

**Affiliations:** Royal Brompton and Harefield Hospitals, Guy’s and St Thomas’ NHS Foundation Trust, London, UK; Royal Brompton and Harefield Hospitals, Guy’s and St Thomas’ NHS Foundation Trust, London, UK; Royal Brompton and Harefield Hospitals, Guy’s and St Thomas’ NHS Foundation Trust, London, UK; Royal Brompton and Harefield Hospitals, Guy’s and St Thomas’ NHS Foundation Trust, London, UK; National Heart and Lung Institute, Imperial College London, London, UK; Royal Brompton and Harefield Hospitals, Guy’s and St Thomas’ NHS Foundation Trust, London, UK; National Heart and Lung Institute, Imperial College London, London, UK

**Keywords:** Cardiac magnetic resonance, Cardio-oncology, Thrombus, Cardiac mass, Case series

## Abstract

**Background:**

Patients with cancer are at an increased risk of thrombus formation, often identified on routine echocardiogram in the right atrium. The 2022 ESC Guidelines on Cardio-oncology emphasize cardiac magnetic resonance (CMR) as the gold standard for thrombus identification.

**Case summary:**

We present a case series of seven patients who underwent CMR due to right atrial mass suspected to result from central venous catheter–related right atrial thrombus. In all cases, CMR enabled accurate diagnosis of a thrombus. It also allowed to assess complete or partial resolution of the thrombi following anticoagulation on follow-up studies.

**Discussion:**

The presence of a central venous catheter is recognized as a risk factor for thrombus formation, particularly when inappropriately advanced into the right atrium. The integration of CMR into the diagnostic pathway enabled precise thrombus identification and guidance for treatment in this population with a complex balance between cancer-related thrombotic and haemorrhagic risks.

**Learning points**

- Utilization of cardiac magnetic resonance (CMR) imaging in cancer patients with suspected thrombi in the right atrium, especially in cases involving central venous catheters, allows for precise identification of thrombi.
- CMR plays a pivotal role in guiding treatment decisions by providing accurate diagnostic information, particularly in patients facing the intricate balance of cancer-related thrombotic and haemorrhagic risks.

## Introduction

Patients with malignancies are at an increased risk of thrombus formation, due to the prothrombotic characteristics of cancer itself, certain cancer treatments, and the use of central venous catheters, often necessary for the periodic administration of chemotherapy. Central venous catheter–related right atrial thrombus (CRAT) is an uncommon yet potentially life-threatening complication. Echocardiogram may initially identify a right atrial (RA) mass, but it cannot definitively distinguish between tumour and thrombus. Cardiovascular magnetic resonance (CMR) offers precise tissue characterization for accurate diagnosis.^1,2^ However, there remains a lack of comprehensive studies specifically addressing the role of CMR in diagnosing CRAT.^3^ We present a case series of seven patients who underwent CMR due to RA mass suspected to result from CRAT (see Supplementary material online, Table S1). In all cases CMR enabled accurate diagnosis of a thrombus. It also allowed for confirming complete or partial resolution following anticoagulation on follow-up studies. This case series presents a useful guide to help clinicians differentiate between thrombus and tumour using CMR.

## Summary figure

Timeline of two cases of central venous CRAT diagnosed by cardiac magnetic resonance.

**Figure:**
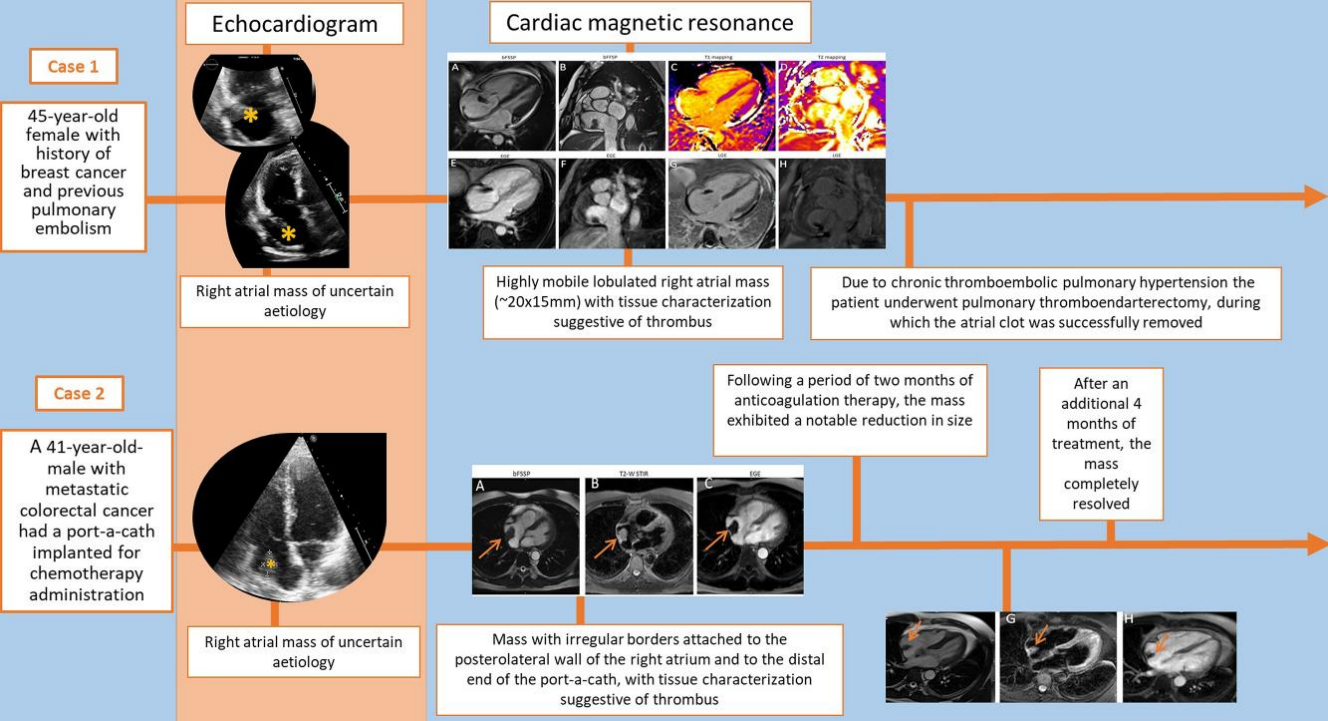


## Case presentation

### Patient 1

**Figure 1:**
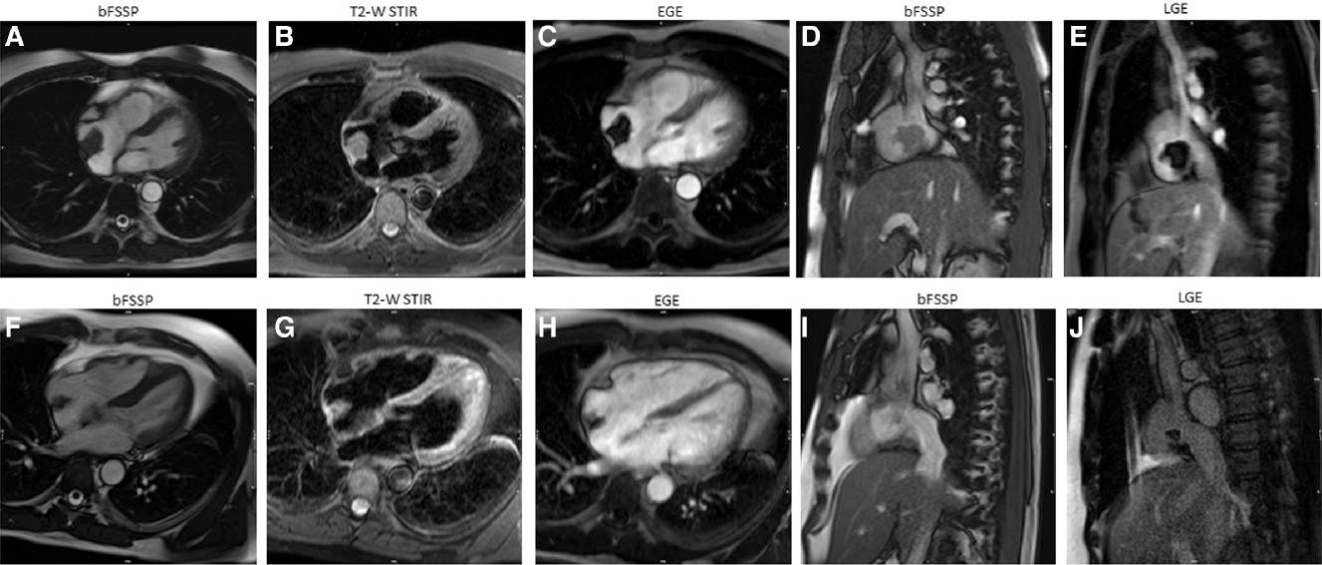
Patient 1.

A 41-year-old-male with metastatic colorectal cancer had a port-a-cath implanted for chemotherapy administration. After detection of a RA mass by echocardiogram, he underwent CMR, which confirmed a mass with irregular borders attached to the posterolateral wall of the RA and to the distal end of the port-a-cath, on balanced steady-state free precession sequence (b-SSFP) cines (*Figure 1A* and *D*). Cardiovascular examination was unremarkable, except for sinus tachycardia. The mass was isointense to the myocardium on T2 short tau inversion recovery (STIR) images (*Figure 1B*). Post-gadolinium enhancement sequences are often the most helpful in confirming the thrombotic nature of the mass. After contrast administration, early gadolinium enhancement images can be acquired with prolonged inversion time (i.e. 600 ms) to selectively null avascular thrombus (*Figure 1C*). In the conventional late gadolinium enhancement sequences, the thrombus also appears as a hypointense structure (*Figure 1E*). Following a period of 2 months of anticoagulation therapy, the mass exhibited a notable reduction in size (*Figure 1F–J*). Subsequently, after an additional 4 months of treatment, the mass completely resolved.

### Patient 2

**Figure 2:**
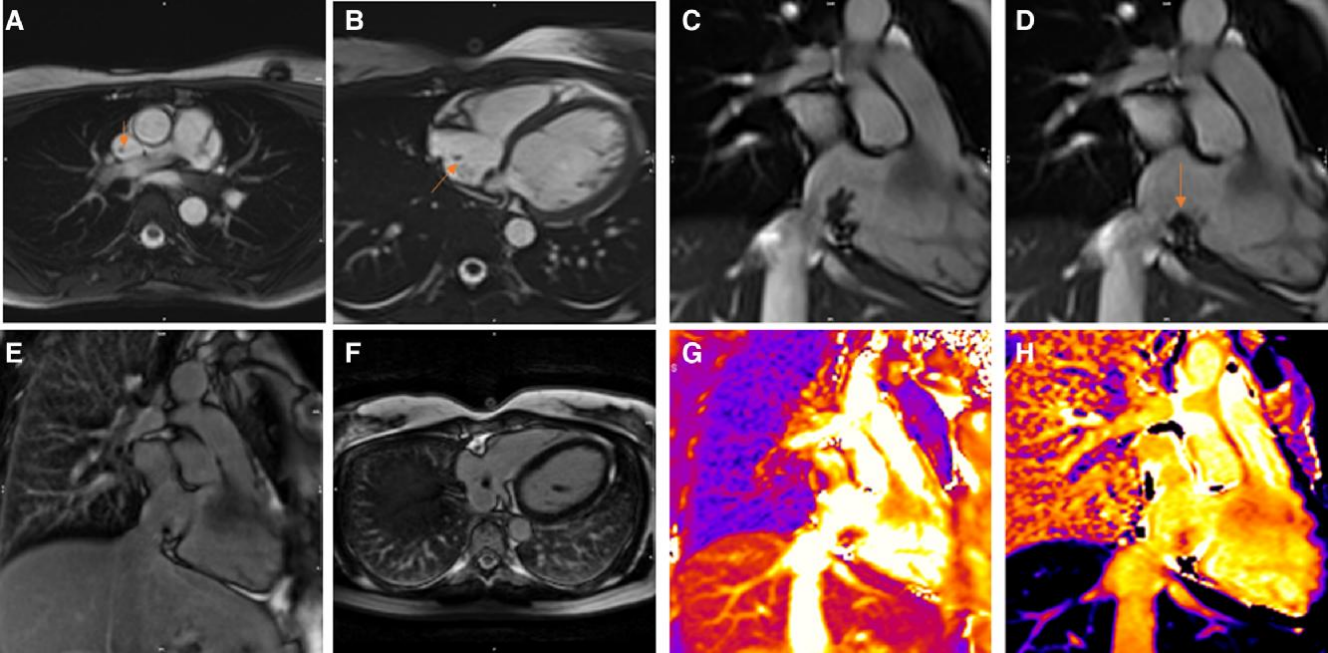
Patient 2.

In the context of breast cancer chemotherapy, a 47-year-old woman underwent the insertion of a port-a-cath. The catheter tip extended to the middle of the RA (*Figure 2A* and *B*—arrow). An echocardiogram performed for ejection fraction monitoring showed a 16 × 10 mm mobile, homogeneously echogenic mass, with irregular borders, adjacent to the Eustachian valve in the inferoposterior RA wall at the tip of the port-a-cath (*Figure 2C* and *D*—arrow). Cardiovascular examination was unremarkable. T1 and T2 mapping are now part of most centre’s protocols for mass evaluation. Compared with myocardium, T2 values are expected to increase in the case of thrombus, while T1 values may be normal to low in case of recent thrombus and normal to high in case of old thrombus.^4^ In this case, the T1 value was decreased compared with the myocardium (885 ms at 1.5T; *Figure 2G*), and the T2 value increased (96 ms at 1.5T), suggesting a recent thrombus (*Figure 2H*). Late post-gadolinium sequences showed no enhancement (*Figure 2E* and *F*). Follow-up CMR after 1 month of anticoagulation revealed a significant decrease in the size of the thrombus.

### Patient 3

**Figure 3:**
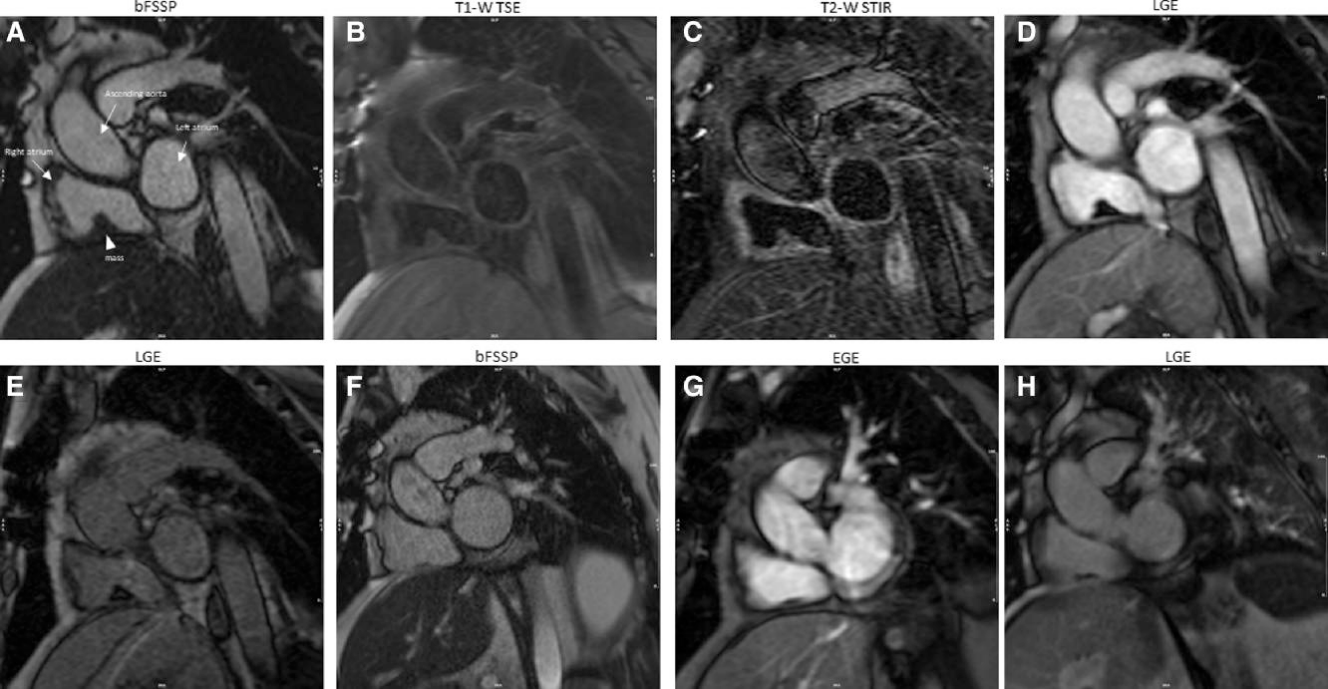
Patient 3.

A 47-year-old male with acute lymphocytic leukaemia had a peripherally inserted central catheter (PICC) for chemotherapy administration. The patient presented with antibiotic refractory fever and persistent raised inflammatory markers. Cardiovascular examination was unremarkable. The PICC line was previously removed with isolation of *Staphylococcus pasteuri*. Transoesophageal echocardiogram was performed to rule out endocarditis and demonstrated a RA mass. CMR was requested for further characterization. Cine b-SSFP images (*Figure 3A*—arrowhead) showed a wide-based non-mobile mass along the inferior wall of the RA. It was isointense to the myocardium on T1-weighted (W) images (*Figure 3B*) and slightly hypointense on T2-W imaging (*Figure 3C*), demonstrating no enhancement on early (*Figure 3D*) and late (*Figure 3E*) post-gadolinium sequences. Overall, tissue characterization was highly suggestive of thrombus. Follow-up CMR demonstrated a significant reduction in the RA mass size after 6 months of anticoagulation (*Figure 3F–H*).

### Patient 4

**Figure 4:**
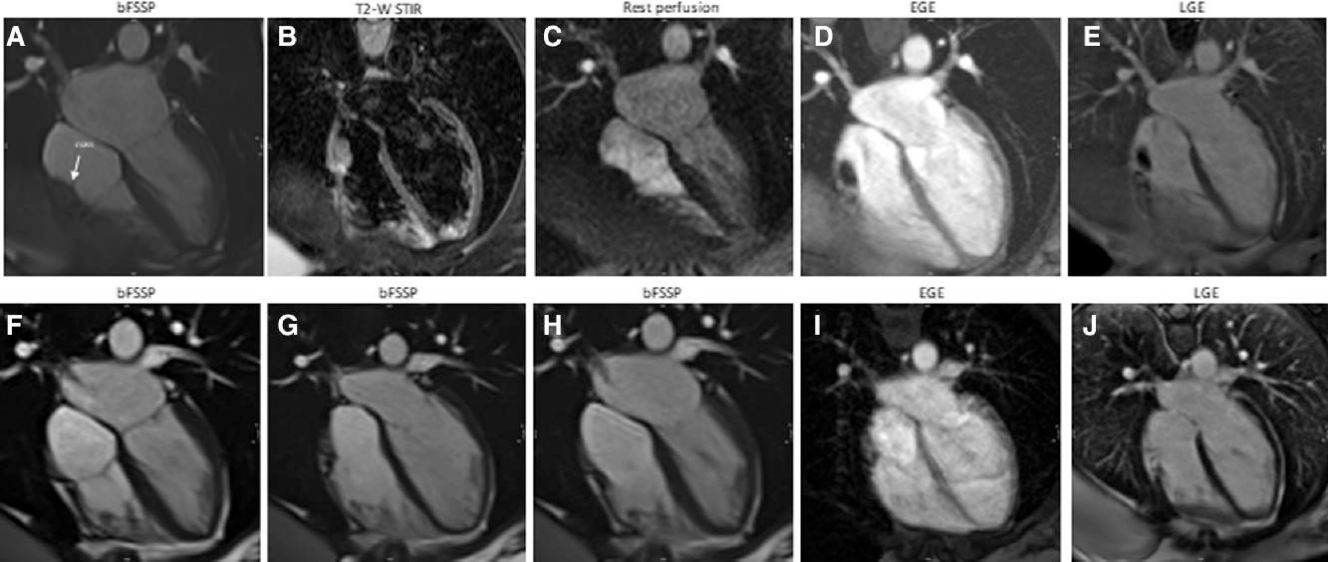
Patient 4.

A 41-year-old-female with breast cancer had a port-a-cath implanted for chemotherapy administration. A RA mass was identified on echocardiogram performed for ejection fraction monitoring. Cardiovascular examination was unremarkable. A subsequent CMR demonstrated the mass being attached to the lateral and inferior aspects of the RA extending to the tricuspid annulus posteriorly on b-FSSP cine (*Figure 4A*). The mass was hyperintense on T2-W (*Figure 4B*) images with no enhancement in early (*Figure 4D*) and late post-gadolinium images (*Figure 4E*). Similarly, no enhancement occurred during first-pass perfusion (*Figure 4C*). Follow-up CMR after 6 months of anticoagulation showed full resolution of the thrombus (*Figure 4F–J*), highlighting the superiority of CMR compared with echocardiogram during treatment response.

### Patient 5

**Figure 5:**
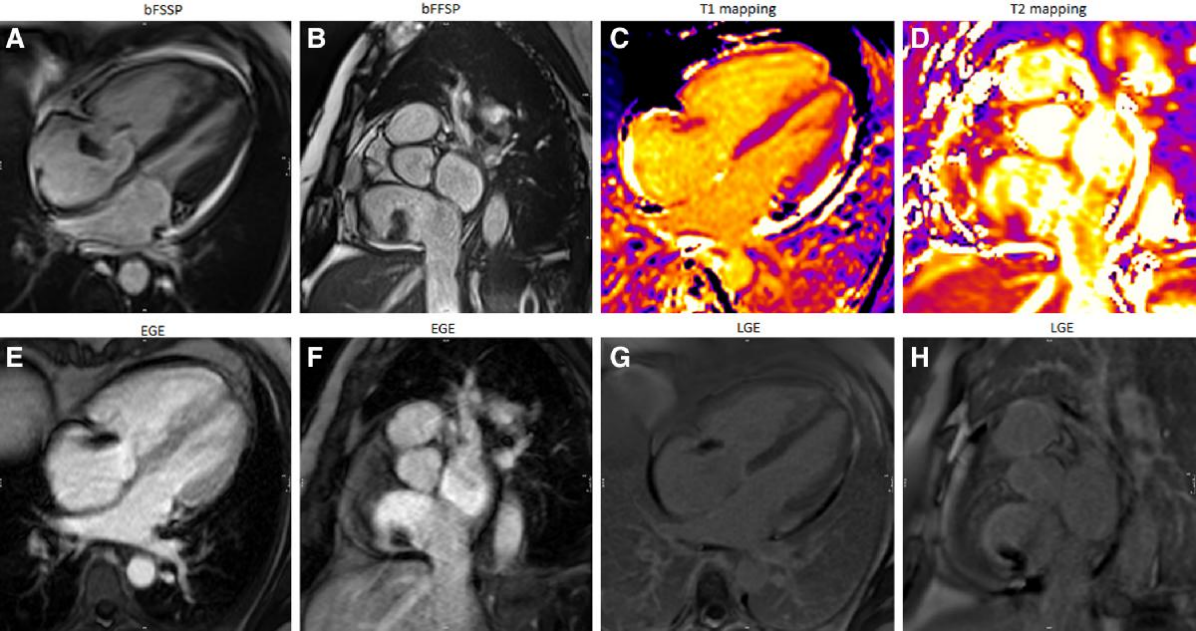
Patient 5.

A 45-year-old female with history of breast cancer and previous pulmonary embolism presented with worsened hypoxaemia and elevated d-dimers. Cardiovascular examination revealed a systolic murmur at the left sternum border and sinus tachycardia. Computed tomography was performed showing bilateral pulmonary embolism. Echocardiogram revealed a RA mass of uncertain aetiology. To further investigate and differentiate between thrombus and malignancy, a CMR was performed. Cardiac magnetic resonance showed a highly mobile lobulated RA mass (∼20 × 15 mm) attached to the RA inferior wall ∼8 mm away from RA/inferior vena cava junction (*Figure 5A* and *B*). T1 mapping values were increased when compared with the myocardium (1192 ms at 1.5T; *Figure 5C*), while T2 mapping values were similar to those of the myocardium (44 ms at 1.5T; *Figure 5D*). There was no enhancement of the mass on the first-pass perfusion study indicating avascularity and, similarly, no enhancement on both early (*Figure 5E* and *F*) and late post-gadolinium sequences (*Figure 5G* and *H*). The patient was diagnosed with chronic thromboembolic pulmonary hypertension and underwent pulmonary thromboendarterectomy, during which the atrial clot was successfully removed. This surgical intervention confirmed the initial CMR diagnosis of a clot within the RA.

### Patient 6

**Figure 6:**
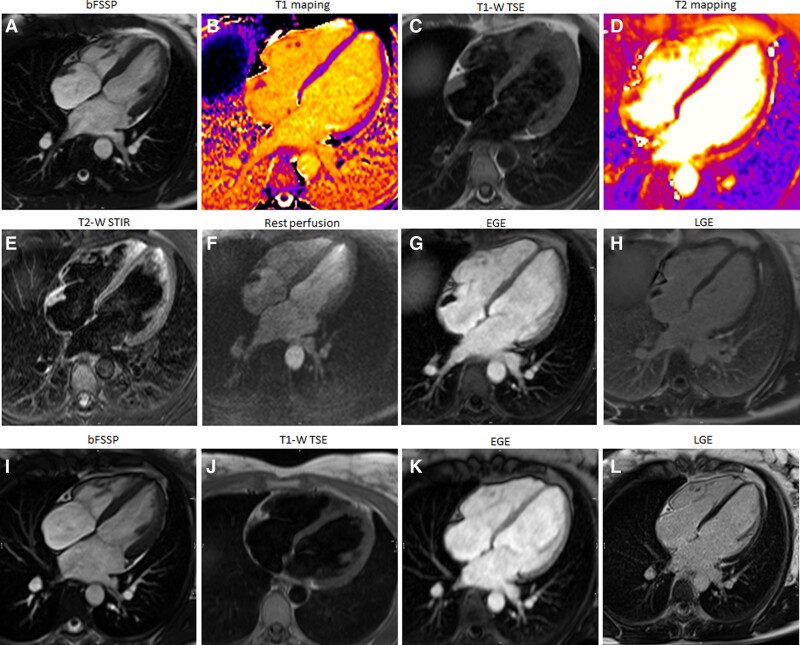
Patient 6.

A 33-year-old female with left breast cancer underwent implantation of a port-a-cath for chemotherapy administration. Surveillance echocardiography revealed a RA mass. Cardiovascular examination was unremarkable. Cardiac magnetic resonance revealed a small sessile mass (∼20 × 15 × 8 mm) attached to the inferolateral RA wall on b-SSFP cines (*Figure 6A*). The mass displayed isointensity when compared with the myocardium in T1 mapping (1064 ms at 1.5T; *Figure 6B*) and T1-W Turbo spin echo (*Figure 6C*) and T2 mapping (50 ms at 1.5T; *Figure 6D*) and T2-W STIR images (*Figure 6E*). There was no observable enhancement in the early post-contrast images (*Figure 6G*) or in the first-pass perfusion sequences (*Figure 6F*) indicating avascularity. Additionally, there was no enhancement of the mass in the late enhancement images (*Figure 6H*). Low position of the port-a-cath tip in the RA was noted. Overall, CMR findings were compatible with a mural thrombus, and follow-up echocardiography and a subsequent CMR scan demonstrated complete resolution of the thrombus following the initiation of anticoagulation therapy (*Figure 6I–L*).

### Patient 7

**Figure 7:**
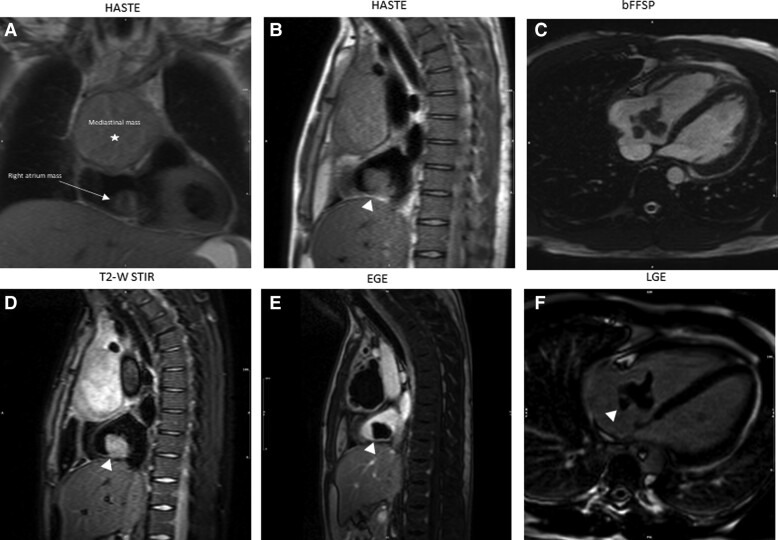
Patient 7.

A 22-year-old male, diagnosed with a mediastinal germ cell tumour, presented with dizziness and palpitations. Initial evaluation revealed a heart rate of 140 b.p.m. and normal blood pressure, and a systolic murmur was heard at the left sternum border. The patient was started on esmolol and anticoagulation, with successful frequency control. Echocardiogram was later performed and revealed a mass in the RA. Cardiac magnetic resonance showed a mediastinal mass (∼68 × 112 × 116 mm) invading the right anterolateral aspect of the heart (*Figure 7A—star*). Additionally, a separate oval-shaped mass was identified in the posterior wall of the lower superior vena cava (SVC), along with mobile pedunculated masses within the RA cavity, attached to the posterior RA wall, seen on b-FSSP cines (*Figure 7A—arrow,*  *Figure 7B—arrowhead,* and *Figure 7C*). The tip of the central catheter was in the upper SVC. The mass was hyperintense to the myocardium on STIR images (*Figure 7D*). While the RA masses displayed the characteristic absence of enhancement observed in thrombi during both early (*Figure 7E*) and post-gadolinium sequences (*Figure 7F*), a definitive distinction from metastases proved challenging, given that the primary tumour also lacked central enhancement. A follow-up study, after anticoagulation, revealed a significant increase in the size of the RA mass, making the diagnosis of metastasis more likely. During the surgical resection of the tumour, it was noted that the mass in the RA was in fact an organized thrombus.

## Discussion

The 2022 ESC Guidelines on Cardio-oncology emphasize the crucial role of CMR in detecting intracardiac thrombus among patients with malignancies. Cardiac magnetic resonance, especially with early and late post-gadolinium sequences, stands out as the gold standard for thrombus identification in this context (*Table 1*).^3,5,6^ In our case series, CMR proved pivotal in accurately diagnosing thrombus in all seven cases.

**Table 1:** Magnetic resonance imaging features of thrombus

	T1-weighted	T2-weighted	T1 mapping values	T2 mapping values	First-pass perfusion	EGE	LGE
Recent thrombus	Isointense to high	High	Normal to low	Increased	No uptake	No uptake	No uptake
Old/chronic thrombus	Isointense to low	Isointense to low	Normal to high	Normal to low	No uptake	No uptake	No uptake

T1-W and T2-W imaging signal is given relative to the myocardium. EGE, early gadolinium enhancement; LGE, late gadolinium enhancement.

The prothrombotic tendencies linked with cancer, alongside the effects of cancer treatments and central venous catheter usage, can contribute to the formation of intracardiac thrombus. Notably, the positioning of the catheter tip within the RA, rather than in the distal SVC, is an identifiable risk factor.

Patients undergoing cardiotoxic anticancer treatments frequently undergo echocardiographic surveillance for left ventricular systolic function, allowing for the early detection of cardiac masses before symptoms, notably embolization, manifest. However, echocardiogram cannot accurately differentiate between thrombus and malignancy. The differential diagnosis is crucial due to its impact on prognosis and cancer treatment, as well as the eventual need to initiate anticoagulation. This is particularly important in a population that not only has a high thrombotic risk but also carries an increased risk of haemorrhage.^5^

Choosing the appropriate anticoagulation should consider thromboembolic and bleeding risks, as well as potential drug-to-drug interactions with anticancer treatments. Novel oral anticoagulants are now viable options for patients with cancer without any of the following bleeding risk factors: unoperated gastrointestinal (GI) or genitourinary (GU) malignancies, history of recent bleeding or within 7 days of major surgery, significant thrombocytopaenia (platelet count < 50 000/µL), severe renal dysfunction (creatinine clearance < 15 mL/min), or GI comorbidities. These risk factors, in turn, favour the use of low-molecular-weight heparin. Anticoagulation should be continued for at least 3 months following catheter removal and until follow-up cardiac imaging confirms thrombus resolution. In cases where the catheter remains in place, long-term anticoagulation is recommended.^4^

This case series highlights the indispensable role of CMR in identifying intracardiac thrombi among cancer patients, particularly following the detection of a mass in transthoracic echocardiography or transoesophageal echocardiography. The integration of CMR into the diagnostic pathway not only ensures precise thrombus identification but also guides treatment in this population with a complex balance between cancer-related thrombotic and haemorrhagic risks.

## Supplementary Material

ytae296_Supplementary_Data

## Data Availability

Data available on request.

## Lead author biography

CMR Fellowship Royal Brompton Hospital General Cardiology Hospital Santa Marta, Lisbon, Portugal

## Notes

**Consent:** The authors confirm that consent for submission and publication of this case series including images and associated text has been obtained from the patients in line with COPE guidance.

**Funding:** None declared.
