# Supplementary material for: Central venous catheter–related right atrial thrombus in oncology patients: a case series of cardiovascular magnetic resonance studies
Source: Eur Heart J Case Rep. 2024 Jun 28;8(7):ytae296. doi: 10.1093/ehjcr/ytae296 (PMC11237891; doi:10.1093/ehjcr/ytae296)
Supplement: ytae296_Supplementary_Data [file ytae296_supplementary_data.docx]

|  | Catheter location | Thrombus directly related to CVC | Follow-up CMR |
| --- | --- | --- | --- |
| Case 1 | Right atrium | Yes | Reduction in size  (2months)  Resolution  (4 months) |
| Case 2 | Removed | No | Reduction in size (1 month) |
| Case 3 | Right atrium | Yes | No change (1 month), reduction (6 months) |
| Case 4 | Right atrium | Yes | Resolution (6 months) |
| Case 5 | - | - | Surgical removed |
| Case 6 | Right atrium | Yes | Resolution  (6 months) |
| Case 7 | Superior vena cava | No | **-** |

**Table 1 – Summary of the 7 cases**
